# The Right Road to Reform

**Published:** 1919-03-15

**Authors:** 


### THE INFLUENCE OF WOMEN WORKERS.

### The Right Road to Reform.

THE influence of women in the public work of the country has increased with startling rapidity in the last year or two. Not only have women obtained the Parliamentary franchise, which is really only one small item in the change, but for the first time they are taking their proper place where anything is being done for the improvement of social conditions. The value of their opinion is everywhere recognised.

How was this change brought about? First, it was *not* brought about by the very silly methods which were tried by some women before the war—putting bombs into public buildings, breaking windows, interrupting church services and meetings, and otherwise making themselves look foolish. Wide opportunities of public usefulness, and a high position in the public service have been opened to women, because they showed during the war that they were thoroughly fit for responsibility and were capable, sensible, and thoughtful, in their work for the general good.

What is true of women in general is true of the nursing profession in particular. During the war nurses have deserved well of their country. The profession has never been more respected and admired than it is now. And with this increase of respect comes a desire in the mind of every one to improve the position of nurses—to give them shorter hours, better salaries, more comforts—and also to help them to raise their professional status by endowing the College of Nursing. All these things are in a fair way to be done, if nothing happens to lower the high esteem which is felt for nurses.

But there is a section, though not a very large one, of the profession which will defeat these ends, if nurses listen to its somewhat raucous and noisy voice. We hear of a trade union for nurses, of strikes, and "down tools." At the meeting held in Dublin to promote a trade union for nurses, one speaker said that nurses were "ground down," and "had opinions imposed on them." Is this the way in which a great profession should be spoken of? It is refreshing to read that the nurse who spoke of her paramount duty to her patient was received with applause, and that several others objected to the idea of a society that could compel them to strike. It is easy to say, as one speaker did, that a strike of nurses is unthinkable. If a union is formed, it must be taken seriously. The final weapon of trade-unionism is the strike. If nurses belong to a union, they must be supposed to be ready to use that weapon. If they do not really intend to do so, they reduce themselves to the level of the foolish mother, who tells her child that she will give him to the sweep if he is not good—both she and the child knowing well that she will do nothing of the sort. Could there be a weaker position?

Unfortunately, the women who suggest these unworthy methods have not the excuse of youth and inexperience of the world. This makes their speeches dangerous, because naturally they carry more weight with nurses. The general public also, though surprised that nurses should speak contemptuously of members of their own profession, will think that they must be well informed, and will believe such statements as that "nurses have no opinions of their own," but accept those "imposed on them" by others. It is perfectly true that many reforms are urgently wanted. But what advantage would it be to us to have short hours and large salaries, if in order to gain them we lost our own self-respect, and the respect of others for us?

"What have you to suggest?" was the very sensible question asked at the Industrial Conference in answer to a somewhat severe criticism of present methods. In this case, however, the reply is easy. If every trained nurse became a really active member of the College of Nursing, we should have a great independent professional body, able to speak with real authority about nurses' desires and needs. Abuses would then be done away much sooner, and conditions of work would quickly improve, because a College representing the whole body of trained nurses would be heard and attended to, and would be able to attain its objects in a proper and constitutional way. We take much too low a view, both of our own influence and of the intelligence and goodwill of the hospital authorities and the public, if we think that they are unwilling to listen to proposals for reform. To get rid of old abuses, and to put things on a better footing, must take time; but with a little perseverance, we shall see reforms carried out.

The respect which is felt for any profession depends upon its own members. No one else can increase or lessen it. At present, in spite of some complaints of individual nurses, the public—that is, our patients and their friends—do trust us. They believe that we shall put our patients' good before all other considerations, and that we shall never refuse our help in time of need. Let us do and say nothing that will lessen that confidence.

Nurses are as well able as other people to manage their own affairs. If we act together as a united profession, we shall be able to gain all the improvements which we rightly desire, without losing our proper status, and, what is of immeasurably greater importance, without losing the confidence and affection of our country, our patients, and our friends.

